# Supplementary material for: History of the Wastewater Assessment of Polio and Non-Polio Enteroviruses in the Slovak Republic in 1963–2019
Source: Viruses. 2022 Jul 22;14(8):1599. doi: 10.3390/v14081599 (PMC9331368; doi:10.3390/v14081599)
Supplement: Supplementary file 1 [file viruses-14-01599-s001.zip › Final Methods Supplementary Table S1.pdf]

Supplementary Table S1. Overview of methods used for wastewater (WW) treatment and cell cultures used for isolation of EVs from WW in the Slovak Republic from 1963 to the present date.

**Period**	**Methods used for sample processing**	**Volume of waste water processed**	**Period**	**Cell cultures used for isolation EVs**
1963-1968	the concentration method included extraction with chloroform	1-5 litres	1962-1990	mainly - monkey kidney cells and the human diploid embryonic lung cells (LEP)<br><br>but also - HeLa cells, chicken fibroblasts, buffalo green monkey kidney (BGM) cells and human amniotic cells
1968-1969	the concentration method included extraction with chloroform, with added aluminium hydroxide	1-5 litres		
1970-1986	the concentration method included extraction with chloroform	1-5 litres		
1986-1994	the flocculation method with the use of aluminium sulphate	10 litres		
1994-1998	the flocculation method with the use of aluminium sulphate and the two-phase separation method with dextran and polyethylene glycol (PEG) (in NRC for poliomyelitis	1-5 litres<br><br>1 litre	1991-1996	cell lines human rhabdomyosarcoma-derived (RD) and human epidermoid carcinoma (Hep2)
1999-present day	the two-phase separation method with dextran and polyethylene glycol (PEG)	1 litre	1997-present day	cell lines human rhabdomyosarcoma-derived (RD), human epidermoid carcinoma (Hep2) and genetically modified mouse cell lines with the human poliovirus receptor (L20B)
